# The Elderly-Nutrient Rich Food Score Is Associated With Biochemical Markers of Nutritional Status in European Older Adults

**DOI:** 10.3389/fnut.2019.00150

**Published:** 2019-09-11

**Authors:** Charlotte S. Kramer, Maria K. Szmidt, Ewa Sicinska, Anna Brzozowska, Aurelia Santoro, Claudio Franceschi, Lisette C. P. G. M. de Groot, Agnes A. M. Berendsen

**Affiliations:** ^1^Division of Human Nutrition and Health, Wageningen University & Research, Wageningen, Netherlands; ^2^Department of Human Nutrition, Warsaw University of Life Sciences–SGGW, Warsaw, Poland; ^3^Department of Experimental, Diagnostic and Specialty Medicine (DIMES), Alma Mater Studiorum, University of Bologna, Bologna, Italy; ^4^Department of Applied Mathematics, Institute of Information Technology, Mathematics and Mechanics (ITMM), Lobachevsky State University of Nizhny Novgorod-National Research University (UNN), Nizhny Novgorod, Russia

**Keywords:** nutrient density, diet quality, micronutrients, status markers, elderly, Europe, health, NU-AGE

## Abstract

**Background:** In order to prevent age-related degenerative diseases in the aging population, their diets should be nutrient dense. For this purpose, the Elderly-Nutrient rich food (E-NRF7.3) score has been developed to assess nutrient density of diets by capturing dietary reference values for older adults. To demonstrate its practical importance such score should be validated against markers of nutritional status and health.

**Objective:** The objective of this study was to examine the association between the E-NRF7.3 score and markers of nutritional status and inflammation.

**Design:** This study was carried out in a sample of the NU-AGE study including 242 Dutch and 210 Polish men and women, aged 65–79 years. Dietary intake was assessed by means of 7-day food records and structured questionnaires collected data on supplement use, lifestyle, and socio-economic information. Baseline measurements included anthropometrics, physical and cognitive function tests, and a fasting venipuncture. E-NRF7.3 scores were calculated to estimate nutrient density of foods and the diet. Associations between the E-NRF7.3 scores and micronutrient status of vitamin D, folate, vitamin B12, homocysteine, and c-reactive protein (CRP) were examined using linear regression analysis while adjusting for confounders.

**Results:** Each one unit increase in E-NRF7.3 score was associated with a 2.2% increase in serum folate in Dutch and 1.6% increase in Polish participants in the fully adjusted models (both *p* < 0.01). Each one unit increase in E-NRF7.3 was significantly associated with a 1.5% decrease in homocysteine levels in Dutch participants (*p* < 0.01), whereas, a 0.9% increase in vitamin B12 levels was observed in Polish participants only (*p* < 0.01). Higher E-NRF7.3 scores were not associated with vitamin D or CRP levels. Adjustment for potential confounders did not substantially alter these results.

**Discussion:** The E-NRF7.3 was developed to reflect dietary intake of relevant nutrients for older adults. Its association with markers of nutritional status could be confirmed for folate (both populations), vitamin B12 (Poland only), and homocysteine (the Netherlands only). There was no association with vitamin D and CRP. To further demonstrate its validity and practical implication, future studies should include a wider range of nutritional status makers, health outcomes, and inflammation markers.

## Introduction

The increasing number of older adults and accompanying age-related degenerative diseases necessitate preventive strategies to lower the disease burden. A healthful diet and adequate nutrient intake could be important strategies to prevent degenerative diseases. However, it is known that there is a high prevalence of inadequate intake of beneficial nutrients on the one hand, with high intakes of nutrients with detrimental effects on health on the other hand (1–4). With decreasing energy needs and increasing nutrient needs for some nutrients, diets of elderly should be nutrient dense (5, 6). Nutrient dense diets can be achieved by means of selecting nutrient-dense foods and beverages to meet nutrient goals without exceeding daily energy needs (7).

One frequently studied tool to assess nutrient density of foods and diets is the Nutrient Rich Food (NRF9.3) index, as proposed by Drewnowski and Fulgoni (7). This index has previously been shown to be related to the risk of stroke (8). Recent research, however, has shown that this NRF9.3 might be of limited use specifically studying diets of European older adults, as it lacks relevant nutrients such as vitamin D and folate and uses dietary reference values not targeted to the European older aged population (9, 10).

Therefore, a new nutrient rich food score was developed with the aim to assess nutrient density of diets in European older adults by including dietary reference values that are relevant for the older aged population. The newly developed nutrient rich food score was composed of nutrients that: (1) have been shown to be of inadequate intake in the older aged population (>20%) (11), (2) were defined as nutrients of public health relevance for older adults, and (3) were associated with relevant health outcomes (12). The nutrient rich food score that best predicted adherence to the NU-AGE index, an index assessing adherence to a healthful diet for the aging population (13, 14), was called the E-NRF7.3 score and included protein, dietary fiber, vitamin D, folate, calcium, potassium, magnesium as nutrients to encourage, and saturated fat, total mono- and disaccharides, and sodium as nutrients to limit (15).

While developing the E-NRF7.3 score, previously proposed science-driven rules were followed, namely: (1) the selection of relevant index nutrients and reference amounts, (2) the development of an appropriate algorithm for calculating nutrient density, and (3) the validation of the chosen nutrient profile model against healthy diets (7). However, the E-NRF7.3 score has not been validated yet against markers of nutritional status and health. To demonstrate its practical application, the validity of this score should be studied. Therefore, we aim to assess the validity of the newly developed E-NRF7.3 against markers of nutritional status and inflammation, for the older aged population, in both Northern and Eastern European older adults.

## Materials and Methods

### Study Population

The present study was carried out as part of the NU-AGE project, a dietary intervention study among 1,294 people living in the Netherlands, Poland, Italy, France, and the UK. The NU-AGE study is a 1-year, randomized, parallel trial designed to combat inflammageing by means of a personally tailored Mediterranean-like dietary pattern, targeting dietary recommendations for European people over 65 years of age (NU-AGE diet). The rationale and design of the study have been described previously (13, 16). In short, at baseline and following 1 year intervention, participants completed 7-day food records and structured questionnaires on medical history, current health, and lifestyle factors. Additionally, participants visited the research center for anthropometric measurements, physical performance, and cognitive function tests, and underwent a fasting venipuncture. All participants gave their written informed consent prior to their inclusion in the study. Ethical approval was provided by the Wageningen University Medical Ethics Committee (the Netherlands) and the Bioethics Committee of the Polish National Food and Nutrition Institute (Poland). The trial is registered at clinicaltrials.gov (NCT01754012).

For the present study, we used baseline data of the Dutch and Polish cohort for whom detailed dietary intake data was available consisting of 252 and 259 apparently healthy men and women aged 65–79 years, respectively, who were enrolled between April 2012 and January 2014. Participants who had not completed the 7-day food record (*n* = 23), with an unlikely energy intake of <500 or >3,500 kcal (*n* = 7) and those with missing data on any of the covariates (*n* = 40) were excluded. A total of 242 Dutch and 210 Polish participants were included in the analysis on biochemical markers of nutritional status and inflammation.

### Dietary Intake Assessment

Average food and nutrient intake was assessed by means of 7-day structured and pre-formatted food records including eight meal occasions (before breakfast, breakfast, morning snacks, lunch, afternoon snacks, evening meal, evening snacks, night snacks) referring to the current day. Participants had a face-to-face training to complete the food records and received written instructions about the level of detail required to describe foods and amounts consumed, including the name of food, preparation methods, recipes for mixed foods, and portion sizes. Portion sizes were reported in household measures, based on pictures or measured in gram or milliliters. During a 1-h interview at the participants home (Netherlands) or at the research center (Poland), a trained dietician/research nutritionist reviewed the food record and frequently used household measures were checked to ensure an adequate level of detail in describing foods and food preparation methods. Consumed foods were coded according to standardized coding procedures and translated into nutrients by use of local food composition tables [Nederlands voedingsstoffenbestand, NEVO 2011 (17), in the Netherlands and National Food and Nutrition Institute (18) in Poland].

### Calculation of the Elderly-Nutrient Rich Food (E-NRF) Score

The E-NRF7.3 score is based upon a selection of nutrients relevant for older adults (Table 1). Nutrients to encourage (NR7) include protein, dietary fiber, folate, vitamin D, calcium, magnesium, and potassium. Nutrients to limit (LIM3) comprise saturated fat, sodium, and total mono- and disaccharides. The development of the E-NRF7.3 score has been described in detail elsewhere (15).

**Table 1:** Dietary reference values for selected nutrients used in calculating the E-NRF7.3.

**Nutrient**	**RDV**	**References**
Nutrient-rich components (NR7)		
Protein, g^a^^,^^b^	112.5 (m), 90 (w)	NNR (19)
Fiber, g	35 (m), 25 (w)	NNR (19)
Calcium, mg	1,200	HCNL (20)
Magnesium, mg	350 (m), 300 (w)	EFSA (21)
Potassium, mg	3,500	EFSA (22)
Vitamin D, μg	20	HCNL/NNR (19, 20)
Folate, μg DFE	330	EFSA (23)
Nutrients to limit (LIM3)		
Saturated fat, g	20	EFSA (24)
Sugar, g	90	EFSA (24)
Sodium, mg^c^	2,400	EFSA (24)

*Population Reference Intakes and Adequate Intakes as set by the European Food Safety Authority (EFSA) (21–23, 25–35), the Nordic Council of Ministers (NNR) (19), the Health Council of the Netherlands (HCNL) (20) as well as the labeling Reference Intake values as set by the EFSA (24) were used as Reference Daily Values (RDV). m, men; w, women*.

a *Values equal to 18% EN*.

b *Based on EFSA reference intakes of 2,500 and 2,000 kcal reference intakes for men and women, respectively*.

c *Value derived from salt reference value using a conversion factor of 2.5*.

The calculation of the E-NRF7.3 score comprised several steps similar to calculating the NRF9.3 (10, 17). First, the NR7 and LIM3 scores were calculated for each food item per 100 kcal. Subsequently, these food scores were converted into individual scores by multiplying the scores by the individual 7-day average amount of energy consumed of each item, in 100-kcal units, and then summing these scores for each subject. Next, the individual LIM3 scores were subtracted from the NR7 scores, resulting in the unweighted E-NRF7.3 score. Lastly, the E-NRF7.3 scores were divided by the number of 100-kcal units of the subjects' average total daily energy intake to provide a “weighted average” individual E-NRF7.3 score.

The algorithms used to calculate the E-NRF7.3 score are listed in Table 2 and are based on sums of nutrients where all nutrients were equally weighted (10). The algorithms which combined positive nutrients and nutrients to limit were based on subtracting the negative from the positive sub score (10). Moreover, the scores were calculated per 100 kcal, since this led to the highest percentage of variance accounted for in previous validation studies (36). Higher E-NRF7.3 scores indicate higher nutrient density on a 100 kcal basis.

**Table 2:** Algorithms used to calculate the E-NRF index scores.

**Model**	**Algorithm**	**Comment**
NR7_100kcal_	∑_i_ = 1–7 (Nutrient_i_/RDV_i_) * 100	Nutrient_i_ = content of nutrient i in 100-kcal edible portion; RDV_i_ = recommended daily values for nutrient i
LIM3_100kcal_	∑_i_ = 1–3 (Nutrient_i_/MDV_i_) * 100	Nutrient_i_ = content of limiting nutrient i in 100-kcal edible portion; MDV_i_ = maximum daily values for nutrient i
E-NRF7.3_100kcal_	NR7–LIM3	Difference between sums

*NR7, nutrient-rich score consisting of 7 beneficial nutrients: protein, dietary fiber, folate, vitamin D, calcium, magnesium, and potassium; LIM3, limited nutrient score consisting of three nutrients to limit: saturated fat, sodium, total mono-disaccharides; E-NRF, elderly nutrient-rich foods score*.

### Biochemical Analysis

Fasting blood samples were obtained by venipuncture in the morning at each of the research centers. Blood samples were stored in a cool storage box with a temperature around 7°C and processed within 3 h after collection.

Concentrations of serum vitamin B12 and folate (chemiluminescence) and plasma homocysteine (enzymatic assay) were measured at the laboratory for biochemical analysis of the Nigrisoli hospital in Bologna, Italy, as described previously (37).

Concentrations of total 25-hydroxyvitamin D (25(OH)D) in all serum samples were measured at the laboratory of the Cork Center for Vitamin D and Nutrition Research, Ireland. 25(OH)D was measured by a modified version of the LCMS/MS method that has been described in detail elsewhere (38).

High sensitivity C-reactive protein (CRP) was quantified via ProcartaPlex^TM^ Immunoassay (Thermo Fisher Scientific) according to the manufacturer's instructions, and with an assay sensitivity of 4.39 pg/mL. Analysis was performed using Luminex 200 instrumentation (Luminex Corporation) in all samples (39) at the gut health Institute of the Quadram Institute Bioscience in Norwich, UK.

### Covariates

A standardized general questionnaire was uses to obtain information on smoking status (never, former, current), educational level (years), and medical history (prevalence of diabetes mellitus type II, hypertension, hypercholesterolemia, neurological diseases, osteoporosis, all: yes/no). Physical activity was assessed using the Physical Activity Scale for the Elderly (PASE) questionnaire (40) and expressed as PASE score. Frailty status (zero being non-frail and one being pre-frail) was assessed with a test described by Fried et al. (41). This test combines measures of unintentional weight loss, handgrip strength, gait speed, self-reported exhaustion, and physical activity. Alcohol intake was assessed by means of food records [virtually no alcohol intake (<0.1 gram of alcohol/day), 0–1 standard glass of alcohol per day (0.1–10 g of alcohol/day) and >1 standard glass of per day (>10 g of alcohol)]. Height was measured with a stadiometer to the nearest 0.1 cm. Weight was measured to the nearest 0.1 kg with a calibrated scale while wearing light clothes. Body mass index (BMI) was calculated as body weight divided by squared body height (kg/m^2^). All measures were taken by a trained research assistant.

### Statistical Analyses

Participants were divided into tertiles on the basis of individual weighted E-NRF7.3 scores. Baseline characteristics were compared between tertiles of the E-NRF7.3 score using analysis of variance for continuous variables. For categorical variables the *X*^2^ statistic was used, unless expected cell counts were <5 for more than 20% of cells, then Fisher's exact test was used.

Linear regression analyses was used to examine the association between the individual weighted E-NRF7.3 scores and markers of nutritional status and inflammation, while adjusting for age, gender (model 1), education, BMI, smoking status, physical activity, energy intake, and alcohol intake (model 2). Linear regression analyses were both performed with individual weighted E-NRF7.3 score as continuous predictor and as categorical predictor using tertiles. Nutritional status and inflammation marker data were transformed using the natural logarithm when residuals were otherwise skewed and thus violating model assumptions. For analyses using transformed markers, back transformed marginal means and coefficients (e^β^) are shown for ease of interpretation.

Statistical analyses were carried out using SPSS version 23.0. A two-sided *p* < 0.05 was considered statistically significant.

## Results

General characteristics of the Dutch and Polish populations are presented in tertiles of individual weighted E-NRF7.3 score in Table 3. The mean E-NRF7.3 score was 10.0 ± 5.3 for Dutch and 11.4 ± 7.4 for Polish participants. The Dutch and Polish participants were on average 71.0 ± 4.0 and 71.5 ± 3.8 years old, had a BMI of 25.9 ± 3.6 and 28.1 ± 4.2, completed 12.4 ± 3.6 and 15.2 ± 2.8 years of education and the vast majority did not smoke (96.7 and 93.3%). These characteristics did not differ significantly across tertiles of the E-NRF7.3 score within either country, except for smoking in Dutch participants (*p* = 0.03).

**Table 3:** General characteristics of 242 Dutch and 210 Polish NU-AGE participants across tertiles of the Elderly Nutrient-Rich Food (E-NRF7.3) score.

**Variable**	**Netherlands**					**Poland**				
	**Total**	**T1**	**T2**	**T3**	***p***	**Total**	**T1**	**T2**	**T3**	***p***
E-NRF7.3 mean	10.0 ± 5.3	4.5 ± 2.6	9.9 ± 1.2	15.8 ± 3.3		11.4 ± 7.4	3.8 ± 3.0	10.7 ± 2.1	19.8 ± 4.5	
Range	(−3.6–27.2)	(−3.6–7.9)	(7.9–11.9)	(11.9–27.2)		(−6.1–30.5)	(−6.1–7.2)	(7.4–14.1)	(14.2–30.5)	
*n*	242	81	81	80		210	70	70	70	
Age, years	71.0 ± 4.0	71.0 ± 4.1	70.9 ± 3.8	71.2 ± 4.3	0.85	71.5 ± 3.8	70.9 ± 3.9	71.7 ± 3.9	71.9 ± 3.6	0.26
Women	135 (55.8)	26 (32.1)	49 (60.5)	60 (75.0)	**<0.01**	127 (60.5)	27 (38.6)	43 (61.4)	57 (81.4)	**<0.01**
BMI, kg/m^2^	25.9 ± 3.6	26.0 ± 3.3	26.0 (4.0)	25.6 ± 3.3	0.74	28.1 ± 4.2	28.3 ± 3.8	27.2 ± 4.1	28.8 ± 4.4	0.07
Smoking status										
Never	121 (50.0)	30 (37.0)	43 (53.1)	48 (60.0)	**0.03**	104 (49.5)	31 (44.3)	37 (52.9)	36 (51.4)	0.30
Former	113 (46.7)	46 (56.8)	36 (44.4)	31 (38.8)		92 (43.8)	32 (45.7)	30 (42.9)	30 (42.9)	
Current	8 (3.3)	5 (6.2)	2 (2.5)	1 (1.3)		14 (6.7)	7 (10.0)	3 (4.3)	4 (5.7)	
Education, years	12.4 ± 3.6	12.6 ± 3.8	12.4 ± 3.5	12.1 ± 3.6	0.75	15.2 ± 2.8	15.6 ± 2.8	15.2 ± 2.9	14.6 ± 2.7	0.12
Physical activity, PASE score	137.5 ± 53.1	137.9 ± 54.2	136.3 ± 53.8	138.3 ± 52.1	0.97	125.8 ± 55.8	136.1 ± 62.8	131.6 ± 51.6	109.8 ± 49.5	**0.01**
Pre-frail^a^	52 (21.5)	20 (24.7)	14 (17.3)	18 (22.5)	0.50	66 (31.6)	19 (27.1)	21 (30.4)	26 (37.1)	0.43
Diabetes mellitus II	9 (3.7)	3 (3.7)	3 (3.7)	3 (3.7)	1.00	17 (8.1)	7 (10.0)	5 (7.1)	5 (7.1)	0.65
Hypertension	79 (32.6)	28 (34.6)	28 (34.6)	23 (28.8)	0.66	129 (61.4)	45 (64.3)	44 (62.9)	40 (57.1)	0.66
Hypercholesterolemia	61 (25.2)	23 (28.4)	18 (22.2)	20 (25)	0.66	76 (36.2)	20 (28.6)	26 (37.1)	30 (42.9)	0.21
Neurological disease	3 (1.2)	2 (2.5)	0 (0)	1 (1.3)	0.55	3 (1.4)	2 (2.9)	1 (1.4)	0 (0)	0.78
Osteoporosis	25 (10.3)	7 (8.6)	7 (8.6)	11 (13.8)	0.47	42 (20.0)	11 (15.7)	13 (18.6)	18 (25.7)	0.31
Dietary intake										
Energy intake, kcal	1,900 ± 383	1,993 ± 384	1,950 ± 382	1,757 ± 342	**<0.01**	1,844 ± 537	2,049 ± 548	1,813 ± 507	1,669 ± 492	**<0.01**
Carbohydrates, EN%	42.1 ± 6.0	41.7 ± 5.9	42.2 ± 5.9	42.3 ± 6.4	0.79	51.9 ± 7.2	49.6 ± 7.9	52.1 ± 8.6	53.9 ± 6.1	**<0.01**
Fat, EN%	34.4 ± 5.1	36.3 ± 4.4	33.9 ± 4.8	32.8 ± 5.4	**<0.01**	34.1 ± 5.8	36.6 ± 6.3	33.8 ± 5.1	31.9 ± 5.0	**<0.01**
Protein, EN%	16.1 ± 2.4	14.9 ± 1.9	15.8 ± 2.0	17.7 ± 2.3	**<0.01**	17.4 ± 3.0	15.9 ± 2.7	17.0 ± 2.5	19.3 ± 2.8	**<0.01**
Alcohol										
<0.1 g/day	35 (14.5)	12 (14.8)	11 (13.6)	12 (15.0)	0.07	92 (43.8)	26 (37.1)	25 (35.7)	41 (58.6)	0.05
0.1–10 g/day	86 (35.5)	24 (29.6)	24 (29.6)	38 (47.5)		94 (44.8)	34 (48.6)	36 (51.4)	24 (34.3)	
>10 g/day	121 (50.0)	45 (55.6)	46 56.8)	30 (37.5)		24 (11.4)	10 (14.3)	9 (12.9)	5 (7.1)	

*Values are expressed as mean ± SD or number (percentage within tertile). Bold values are statistically significant. BMI, body mass index; EN%, energy percent; PASE, physical activity scale for the elderly*.

a *Poland: n = 209*.

In both countries, participants with higher E-NRF7.3 scores were most likely to be woman (75% in the Netherlands and 81% in Poland, both *p* < 0.01), had lower energy intake (*p* < 0.01), fat intake (*p* < 0.01), and higher protein intake (*p* < 0.01), compared to participants with lower E-NRF7.3 scores. Polish participants with the highest E-NRF7.3 score had a significantly higher carbohydrate intake (*p* < 0.01) and a lower level of physical activity (*p* = 0.01) compared to those with lower E-NRF7.3 scores.

In both populations, folate levels were significantly higher in the group with highest E-NRF7.3 scores [geometric mean (95% CI): 11.1 (9.6–13.0) vs. 7.9 (6.9–9.1), *p* < 0.01 in the Netherlands and 10.4 (9.1–11.8) vs. 7.8 (6.9–8.8), *p* < 0.01 in Poland, after full adjustment, Table 4]. Continuously, a one unit increase in E-NRF7.3 score was associated with a predicted 2.2% increase in folate levels in Dutch participants (e^β^ = 1.022, *p* < 0.01) and 1.6% in Polish participants (e^β^ = 1.016, *p* < 0.01).

**Table 4:** Association between the Elderly Nutrient-Rich Food (E-NRF7.3) score and markers of nutritional status and inflammation in Dutch and Polish NU-AGE participants.

	**Netherlands**						**Poland**					
	**T1**	**T2**	**T3**		**Continuous**		**T1**	**T2**	**T3**		**Continuous**	
	**mean** **(95% CI)**	**mean** **(95% CI)**	**mean** **(95% CI)**	***p***	**β**	***p***	**mean** **(95% CI)**	**mean** **(95% CI)**	**mean** **(95% CI)**	***p***	**β**	***p***
**Folate^a^**	*n* = 81	*n* = 81	*n* = 80				*n* = 70	*n* = 68	*n* = 70			
Crude	8.7 (7.8–9.5)	9.9 (9.0–11.0)	12.9 (11.7–14.3)	**<0.01**	1.029	**<0.01**	7.5 (6.8–8.2)	10.1 (9.2–11.2)	10.9 (9.9–12.1)	**<0.01**	1.021	**<0.01**
Model 1	8.9 (8.0–9.8)	9.8 (8.9–10.8)	12.5 (11.3–13.8)	**<0.01**	1.024	**<0.01**	7.7 (7.0–8.5)	9.8 (8.9–10.8)	10.1 (9.1–11.2)	**<0.01**	1.015	**<0.01**
Model 2	7.9 (6.9–9.1)	8.8 (7.6–10.2)	11.1 (9.6 – 13.0)	**<0.01**	1.022	**<0.01**	7.8 (6.9–8.8)	9.9 (8.7–11.2)	10.4 (9.1–11.8)	**<0.01**	1.016	**<0.01**
**Vitamin B12^a^**	*n* = 81	*n* = 81	*n* = 80				*n* = 70	*n* = 68	*n* = 69			
Crude	368.9 (343.6–396.1)	377.4 (351.5–405.2)	407.1 (379.0–437.3)	0.13	1.008	0.06	315.2 (293.3–338.6)	363.3 (337.8–390.8)	373.4 (347.3–401.4)	**<0.01**	1.011	**<0.01**
Model 1	373.5 (347.2–401.5)	375.0 (349.1–402.8)	400.2 (371.3–431.2)	0.35	1.005	0.24	318.3 (295.9–342.2)	360.0 (334.3–387.5)	364.7 (337.2–394.4)	**<0.01**	1.009	**<0.01**
Model 2	376.5 (340.7–416.3)	378.8 (340.0–421.7)	397.4 (355.0–445.1)	0.56	1.003	0.50	315.5 (289.1–343.9)	349.0 (318.5–382.2)	359.6 (327.2–395.2)	**<0.01**	1.009	**<0.01**
**Homocysteine^a^**	*n* = 81	*n* = 81	*n* = 80				*n* = 70	*n* = 68	*n* = 70			
Crude	11.8 (11.2–12.5)	10.7 (10.16–11.3)	9.7 (9.2–10.3)	**<0.01**	0.984	**<0.01**	12.4 (11.4–13.6)	11.2 (10.3–12.3)	10.8 (9.9–11.8)	0.07	0.992	**0.02**
model 1	11.7 (11.1–12.4)	10.8 (10.2–11.4)	9.9 (9.3–10.4)	**<0.01**	0.986	**<0.01**	12.2 (11.2–13.4)	11.5 (10.5–12.5)	11.4 (10.4–12.5)	0.45	0.995	0.20
model 2	12.3 (11.4–13.3)	11.4 (10.6–12.4)	10.3 (9.5–11.2)	**<0.01**	0.985	**<0.01**	12.5 (11.3–13.9)	11.9 (10.7–13.3)	11.5 (10.3–12.9)	0.43	0.994	0.12
**Vitamin D**	*n* = 81	*n* = 81	*n* = 80				*n* = 70	*n* = 69	*n* = 70			
Crude	60.9 (56.9 – 65.0)	61.8 (57.8–65.9)	65.3 (61.3–69.4)	0.28	0.381	0.09	52.7 (47.9–57.4)	60.9 (56.2–65.7)	56.6 (51.9–61.4)	0.06	0.059	0.76
Model 1	61.4 (57.2–65.5)	61.5 (57.5–65.6)	64.8 (60.6–69.1)	0.44	0.314	0.20	52.7 (47.8–57.5)	61.0 (56.1–65.8)	56.7 (51.6–61.8)	0.06	0.033	0.88
Model 2	54.9 (49.3–60.4)	54.6 (48.6–60.5)	58.3 (52.1–64.6)	0.39	0.268	0.28	52.6 (46.8–58.5)	61.0 (54.9–67.1)	58.0 (51.7–64.3)	0.06	0.177	0.42
**CRP^a^**	*n* = 78	*n* = 79	*n* = 78				*n* = 69	*n* = 63	*n* = 66			
Crude	1,203,357 (952,375–1,520,482)	1,018,196 (807,030–1,284,615)	920,129 (728,219–1,162,614)	0.27	0.979	0.10	801,207 (618,998–1,037,051)	868,473 (662,959–1,137,696)	850,418 (653,219–1,107,149)	0.75	0.999	0.93
Model 1	1,184,700 (932,499–1,505,708)	1,026,843 (811,636–1,299,410)	942,226 (737,113–1,203,436)	0.43	0.982	0.19	814,231 (626,246–1,058,166)	852,561 (647,905–1,122,311)	815,046 (613,549–1,083,055)	0.96	0.995	0.64
Model 2	1,339,759 (971,465–1,846,871)	1,148,538 (816,613–1,616,621)	1,070,889 (748,961–1,531,167)	0.42	0.984	0.26	931,918 (682,155–1,272,178)	1,057,058 (750,284–1,490,238)	914,379 (648,696–1,289,412)	0.70	0.989	0.33

*Folate was measured in ng/mL, vitamin B12 was measured in pg/mL, homocysteine as μmol/L, vitamin D as ng/mL, CRP as pg/mL. Bold values are statistically significant*.

a *Natural logarithm used, values are exponentiated values of marginal means (geometric mean), and β (e^β^)*.

*Model 1: adjusted for age and sex*.

*Model 2: additionally adjusted for education, BMI, smoking status, physical activity, energy intake, and alcohol intake*.

*95% CI, 95% confidence interval; CRP, c-reactive protein*.

Each 1 unit increase in E-NRF7.3 score was associated with a 0.9% increase in vitamin B12 levels in Polish participants (e^β^ = 1.011, *p* < 0.01 in the crude model and e^β^ = 1.009, *p* < 0.01 in the fully adjusted model). In Dutch participants, vitamin B12 levels were not significantly higher with increasing E-NRF7.3 score.

With higher E-NRF7.3 scores homocysteine levels significantly decreased in both populations [geometric mean (95%CI) 9.7 (9.2–10.3) vs. 11.8 (11.2–12.5), *p* < 0.01 in the Netherlands and 10.8 (9.9–11.8) vs. 12.4 (11.4–13.6), *p* = 0.07 in Poland], with a 0.08% decrease in Poland (e^β^ = 0.992, *p* = 0.02) and 1.6% decrease in the Netherlands (e^β^ = 0.984, *p* < 0.01) for each unit increase in E-NRF7.3 score in the crude model. When adjusting for potential confounders, the association remained significant in the Dutch population only [geometric mean 10.3 (9.5–11.2) vs. 12.3 (11.4–13.3), *p* < 0.01 and e^β^ = 0.985, *p* < 0.01).

For vitamin D, a borderline significant positive association was observed in the Polish population across tertiles of E-NRF7.3 score, in all adjustment models [mean value (95%CI) 58.0 (51.7−64.3) in the highest tertile vs. 52.6 (46.8−58.5) in the lowest tertile, *p* = 0.06], but not per one unit increase in E-NRF7.3 score (β = 0.177, *p* = 0.42 in fully adjusted model).

CRP levels did not differ across tertiles of E-NRF7.3 scores in either the Dutch or Polish population (all *p* > 0.10). Continuously, there was also no association between E-NRF7.3 score and CRP level (e^β^ = 0.984, *p* = 0.26 in the Netherlands and e^β^ = 0.989, *p* = 0.33 in Poland after full adjustment).

## Discussion

The E-NRF7.3 score was developed with the aim to capture nutrient density of foods and diets of older adults by including nutrients that are of relevance for this population. Although the E-NRF7.3 score was shown to be nicely correlated with greater adherence to a healthful diet for the aging population within a Dutch population, it has not been evaluated in relation to markers of nutritional status and inflammation in other populations. In this cross-sectional study, higher E-NRF7.3 scores were significantly associated with higher folate blood levels in both populations, higher vitamin B12 levels in the Polish population, and with lower homocysteine levels in the Dutch population. These results remained after adjustment for energy intake and various lifestyle and personal factors.

Folate and vitamin B12, as well as other B vitamins are essential for the methylation of homocysteine to methionine (42) and are therefore key players in life maintenance via methylation processes and DNA precursors (43). Therefore, high homocysteine levels are the result of low folate and vitamin B12 levels. In turn, high homocysteine levels are associated with increased risk of cardiovascular disease, dementia, stroke, and depression (37, 44–46).

Considering the inclusion of folate equivalents in the E-NRF7.3 score a positive association with serum folate levels can be expected. This is in line with previous analyses of nutrient intakes and blood biomarkers in all five NU-AGE intervention countries by Ostan et al., reporting a significant correlation between folate intake and serum concentrations (ρ = 0.363, *p* < 0.01) (37). A study in Italian and British adults reported similar results, where a 100 μg/d increase in dietary folate intake was associated with a 13.8 and 10.5% increase in serum folate levels, respectively (47).

Interestingly, the E-NRF7.3 score showed a significant positive association with serum vitamin B12 levels in Polish participants whereas the index did not include dietary vitamin B12. Although Ostan et al. observed that vitamin B12 intake significantly correlated with serum concentrations (ρ = 0.151, *p* < 0.01) (37), Jungert et al. found that vitamin B12 intake was not a predictor of serum vitamin B12 status. In their study, serum folate was the main predictor of serum vitamin B12 in healthy community-dwelling older adults (β = 0.407, *p* < 0.01) (48), possibly explaining the association found with the E-NRF7.3 score.

While developing the E-NRF7.3, the inclusion of vitamin B12 was considered as it is an important nutrient for older adults and it is related to relevant health outcomes. However, including vitamin B12 to the E-NRF7.3 reduced the validity instead of improving it. Therefore, vitamin B12 was omitted from the E-NRF7.3 (15). This approach is in line with the extensively studied NRF9.3, for which a threshold for the useful number of nutrients exists, after which the ranking of products or prediction of healthy diet index declined (49, 50). We did include serum B12 in the present study as it was hypothesized that a nutrient dense diet based on nutrients included in the E-NRF7.3 is likely to be nutrient dense for other relevant nutrients that are not included in the E-NRF7.3. The Polish data seem to support this hypothesis, however further studies would be useful.

The positive association of the E-NRF7.3 score with vitamin B12 level and the inverse association with homocysteine level were only significant in the Polish and Dutch participants, respectively, whereas the non-significant associations did show a similar trend. An explanation for the different findings between the countries could be related to varying ranges of vitamin B12 and homocysteine values within countries. In Dutch participants, the range of vitamin B12 in the highest compared to the lowest E-NRF tertile is around 21, whereas the range for Polish participants is 44. For homocysteine levels the opposite is observed with a wider range in Dutch participants (range of 2) compared to Polish participants (range of 1). A wider range in the study population makes detection of a significant association more likely. This could be a reason that significant associations are only shown for the population with the widest range of the biomarker. Additionally, serum vitamin B12 does not show high sensitivity and specificity, so is limited in its use as a marker (51).

Moreover, although both vitamin B12 and folate levels are considered concentration markers of micronutrient status, several physiological and environmental factors other than diet, such as polymorphisms, and certain drugs, also influence their blood levels (52, 53). For homocysteine, renal function influences levels via clearance (54). For vitamin B12, inflammation of the gastric mucosa can cause reduction in the acid required to cleave vitamin B12 from food protein (55). Since the Polish and Dutch participants were very similar regarding age, sex, disease incidence, and macronutrient intakes, perhaps differences in physiological and environmental factors that have not been measured in these populations, such as kidney function or gastric differences, additionally add to differences in associations of the E-NRF7.3 score with B12 and homocysteine (51).

Previous studies on homocysteine level predictors have not included nutrient density scores, however, indices of the Mediterranean diet have been studied. When developing the E-NRF7.3 score its correlation with the NU-AGE index, a Mediterranean-like dietary pattern (13), was considered. Similar to studies in adults observing a negative association between the MedDietScore and homocysteine levels in adults, our study shows an inverse association between the E-NRF7.3 score and homocysteine levels (56, 57). Folate intake has been shown to be negatively associated with homocysteine levels (58), and folate and folic acid lower homocysteine in people with moderate hyperhomocysteinemia (59). Additionally, low vitamin B2, B6, and B12 levels are associated with increased homocysteine levels (43).

Besides an association of the Mediterranean diet with homocysteine, Chrysohoou et al. found that participants in the highest tertile of the Mediterranean diet score had 20% lower CRP levels (56) compared to participants in the lowest tertile. Similarly, a systematic review on dietary patterns and inflammation markers showed that nearly three-quarters of the studies using dietary indices or scores, and especially using the Mediterranean diet score, found negative associations with CRP levels (60). Other studies reported that close adherence to a Mediterranean diet was related to the inflammation marker fibrinogen, but not to CRP concentrations in community-dwelling older adults. However, “health aware” dietary patterns (low-fat and high-fruit) and high fruit intake were inversely associated with CRP (61).

Although, the E-NRF7.3 score is correlated with the NU-AGE diet, which resembles the Mediterranean diet, the E-NRF7.3 score does not include vitamins such as vitamin C and flavonoids mainly found in fruit. Therefore, the components of the Mediterranean diet that possibly result in the negative association with CRP-levels might not be completely captured in the E-NRF7.3 score.

The E-NRF7.3 score was also not significantly associated with vitamin D serum levels, despite the inclusion of vitamin D in the index. In contrast to folate and vitamin B12 levels, vitamin D is not only derived from oral intake, but additionally synthesized in the skin upon ultraviolet-B light exposure. Even in older adults at relatively northern European latitudes, daily ambivalent ultraviolet-B dose contributes significantly to 25(OH)D levels (62, 63). Moreover, a study by Brouwer-Brolsma et al. in Dutch older community-dwelling adults showed that vitamin D intake from foods, supplements, genetics and education, lifestyle and personal characteristics only explained approximately one-third (*R*^2^ = 0.35) of 25(OH) D levels. Similar percentages of 28–33% have been found in by others (64, 65), suggesting that other factors contribute significantly to 25(OH)D variation.

The newly developed E-NRF7.3 score followed specific recommendations as proposed by Drewnowski and Fulgoni (7) by firstly including nutrients that are relevant for the aging population, defined as nutrients that are commonly inadequately consumed by elderly and nutrients that are associated with health outcomes relevant to elderly. Moreover, local nutrient composition databases have been used. Secondly, appropriate reference daily values were used by including the European Food Safety Authority, complemented with reference values more specific to older adults for selected nutrients and labeling reference values for the three nutrients to limit (15). Thirdly, we aimed to keep the algorithm both simple and transparent by adjusting previously developed NRFn and NRFn.3 scores (7, 66). Fourthly, previously the E-NRF7.3 score was validated against the NU-AGE index (15), a measure of adherence to the anti-inflammageing NU-AGE diet (13, 14). The current paper demonstrates its validity against a selection of markers of nutritional status and inflammation.

Strengths of this study include the 7-day food records with a standardized protocol used in both countries. Food records show better association with energy and protein biomarkers than Food Frequency Questionnaires and 24-h recalls (67) and rely less on memory compared to Food Frequency Questionnaires and 24-h recalls since participants record food intake at time of consumption (68, 69). Extensive information on food item level was available for thousands of products per country, as well as a wide range of confounding variables, from diet, physical activity, and anthropometric measurements to alcohol and smoking. An advantage of a nutrient density score is that it does not include foods or food groups that are not consumed as has previously been an issue with dietary indices (70). This allows for use in various regions and countries.

Limitations of this study include differences between national food consumption databases used. For Polish participants sucrose and lactose were used for E-NRF7.3 score calculations, where total mono- and disaccharides were available for Dutch participants. This could have contributed to higher E-NRF7.3 scores for Polish participants, as the monosaccharides in for example fruits and honey did not contribute to the LIM3 part of the score. However, Streppel et al. found that when using the NRF9.3 index in relation to health outcomes, replacing total sugar with added sugar did not alter the results (8). Therefore, the influence of the different sugars used in calculation on the association with biomarkers is likely to be small. Further differences between the countries could result from variability in estimation of the quantity of nutrients in the same food between food composition databases (37) as well as differences in nutrient densities of similar food items resulting from compulsory margarine fortification with vitamin D (among others) in Poland, compared to only voluntary food fortification in the Netherlands (71).

Although, some dietary patterns and single nutrient intakes have been studied in relation to markers of intake and health outcomes, this is the first study demonstrating an association between a nutrient density score specifically developed to capture relevant nutrients for older adults and markers of nutritional status. In reflection of the current results, the addition of more or other nutrients to the E-NRF7.3 score could be considered as a way to further increase its validity with markers of nutritional status. Future studies should study the association with a wider range of health outcomes relevant to European older adults, and more specific markers of chronic inflammation. Furthermore, to demonstrate the practical applicability of the E-NRF7.3 score, this score should be linked to other determinants of food choice, including food preferences, food costs, food enjoyment, and availability (7).

To conclude, we observed that people with higher E-NRF7.3 scores have significantly higher folate levels, higher vitamin B12 levels (Poland) and lower homocysteine levels (Netherlands). Future studies should be undertaken in which more markers of nutritional status, a wide range of health outcomes and the practical implication of the score can be investigated.

## Data Availability

The datasets generated for this study are available on request to the corresponding author.

## Ethics Statement

The studies involving human participants were reviewed and approved by Wageningen University Medical Ethics Committee, the Netherlands Bioethics Committee of the Polish National Food and Nutrition Institute, Poland. The patients/participants provided their written informed consent to participate in this study.

## Author Contributions

AS and CF designed the NU-AGE project. AAMB and LG designed the intervention study. LG, AB, and AS were the principal investigators in the Netherlands and Poland. AAMB and ES conducted the dietary intervention in the Netherlands and Poland. AAMB and MS were responsible for the nutrient intake database in the Netherlands and Poland. CK and AAMB analyzed and interpreted the data and drafted the manuscript. LG interpreted the data. LG, AAMB, CK, ES, AB, AS, and CF critically revised the manuscript for important intellectual content.

### Conflict of Interest Statement

The authors declare that the research was conducted in the absence of any commercial or financial relationships that could be construed as a potential conflict of interest.
